# Thyroid Pneumatosis Following Fishbone Ingestion: A Rare Case Report

**DOI:** 10.1155/crie/4133056

**Published:** 2026-03-03

**Authors:** Bruna Pimentel, Luís Baptista, Inês Medeiros, Beatriz Lança, Ana Coutinho Santos, Clara Cunha, João Sequeira Duarte

**Affiliations:** ^1^ Endocrinology Department, Lisboa Ocidental Local Health Unit, Lisbon, Portugal; ^2^ Otolaryngology Department, Lisboa Ocidental Local Health Unit, Lisbon, Portugal; ^3^ Intensive Care Department, Lisboa Ocidental Local Health Unit, Lisbon, Portugal; ^4^ Radiology Department, Lisboa Ocidental Local Health Unit, Lisbon, Portugal

**Keywords:** destructive thyroiditis, esophageal perforation, retropharyngeal abscess, thyroid pneumatosis, thyrotoxicosis

## Abstract

**Background:**

Thyroid pneumatosis is an extremely rare condition, previously reported only once as a complication of an ultrasound‐guided thyroid fine needle aspiration (FNA).

**Case Report:**

A 70‐year‐old male sought emergency care after experiencing a foreign body sensation in his throat. He was diagnosed with a retropharyngeal abscess caused by upper digestive tract perforation from fishbone ingestion, which was surgically extracted. Postoperative day 3, a neck computed tomography (CT) revealed gas bubbles within the thyroid parenchyma, indicative of thyroid pneumatosis, likely leading to destructive thyroiditis (TSH 0.109 µUI/mL, free T4 of 27.1 pmol/L, and free T3 of 4.08 pmol/L). A multidisciplinary team opted for a conservative approach, and the patient showed a favorable recovery. Thyroid function normalized within 2 months (TSH 2.44 mUI/mL, free T4 13.6 pmol/L, and free T3 4.58 pmol/L).

**Conclusion:**

Thyroid pneumatosis should be recognized as a potential rare complication following esophageal perforation. This is the first documented case linking destructive thyroiditis to thyroid pneumatosis.

## 1. Introduction

This report presents a rare case of thyroid pneumatosis secondary to upper digestive tract perforation caused by fishbone ingestion and subsequent extraction. To the best of our knowledge, the only previously documented case of thyroid pneumatosis was reported as a complication of an ultrasound‐guided thyroid fine needle aspiration (FNA) biopsy in a 44‐year‐old woman [1], and notably, no thyroid dysfunction was observed in this case.

## 2. Case Presentation

A 70‐year‐old male sought emergency care due to a sensation of a foreign body in his throat, which started 3 days after eating fish. He reported no dysphagia, sialorrhea, or dyspnea.

The patient’s medical history was only significant for obesity, dyslipidemia, and prediabetes.

During observation, he was febrile (tympanic temperature of 39°C), hemodynamically stable, and saturating well on room air with no signs of respiratory distress. Inspection of the oropharynx and evaluation with 70° laryngoscopy were unremarkable. The patient did not have cervical tenderness, masses, or subcutaneous emphysema on palpation.

Laboratory findings revealed leukocytosis (16.7 × 10 ^9^/L) with a predominance of neutrophils (83%) and a C‐reactive protein level of 11.7 mg/dL.

A contrast‐enhanced computed tomography (CT) showed a horizontal foreign body measuring 35 mm in length and 3–4 mm in diameter, consistent with a fishbone, within the lumen of the digestive tract immediately above the upper esophageal sphincter, with associated perforation and extensive gas densities on its left lateral aspect. There was densification of the adjacent fat of the left lateral cervical spaces as well as an elongated retropharyngeal abscess measuring 44 × 15 × 65 mm. The airway column was patent.

Under general anesthesia, the esophageal foreign body was completely extracted via rigid esophagoscopy. The retropharyngeal abscess was drained transorally, and no drain placement was required. The patient was admitted to the intensive care unit and initiated amoxicillin‐clavulanate and clindamycin for neck space infection, which was continued for 2 weeks. He remained on invasive mechanical ventilation for airway protection for 24 h. Blood cultures were negative.

**Figure 1:**
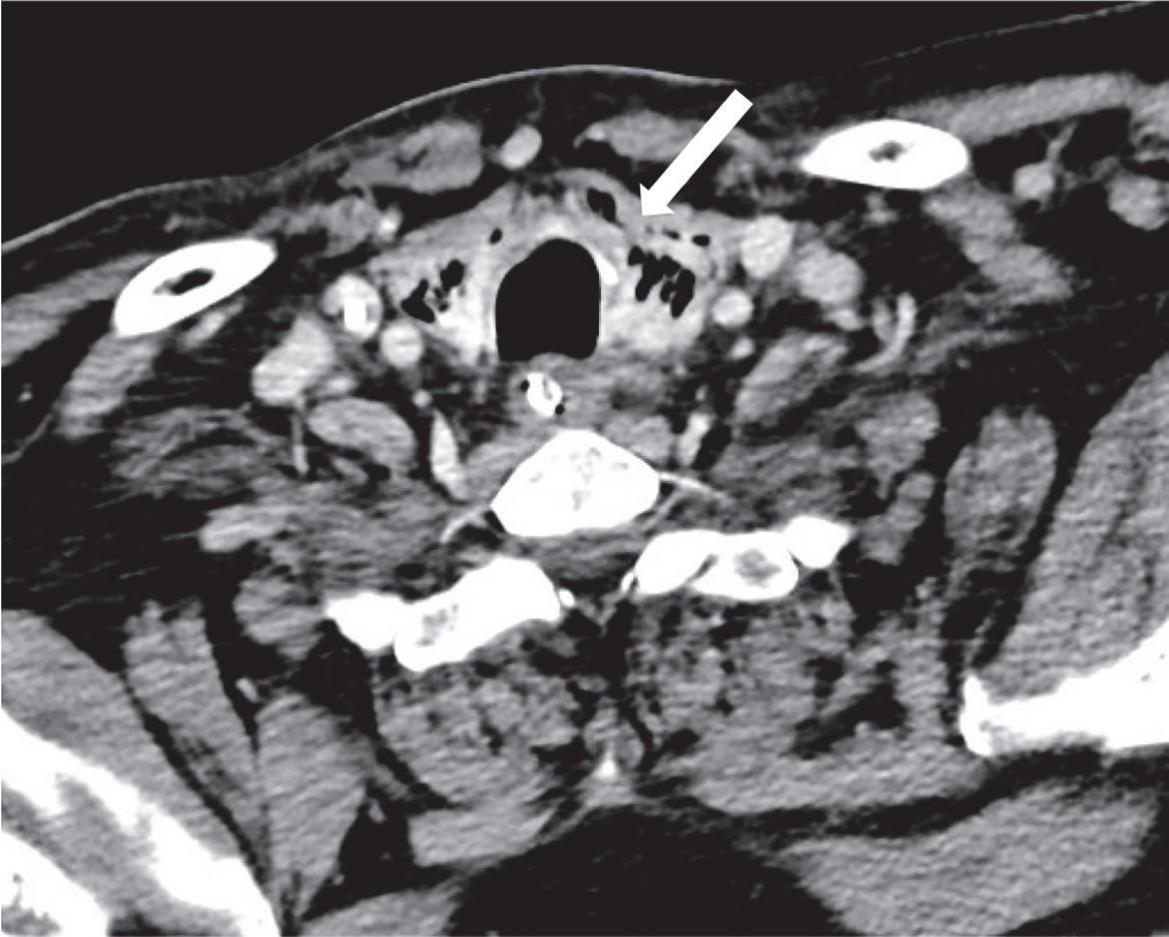
Axial image of IV contrast‐enhanced neck CT (1.50 mm slice thickness, contrast‐enhanced neck CT, venous phase, and 70 s), showing multiple gas bubbles within the thyroid parenchyma (arrow).

Three days after foreign body extraction, a follow‐up contrast‐enhanced neck CT revealed multiple gas bubbles within the thyroid parenchyma, indicating pneumatosis. A gaseous tract with ~3 mm in thickness was defined along the posterosuperior border of the left thyroid lobe (shown in Figure 1) likely extending contiguously from gas bubbles present in a small residual cavity adjacent to the left lateral wall of the larynx. No gas bubbles in the mediastinum or signs of mediastinitis were observed.

At the same time, there was laboratory evidence of thyrotoxicosis with a TSH 0.109 µUI/mL (reference range: 0.27–4.20 µUI/mL), free T4 of 27.1 pmol/L (reference range: 12.0–22.0 pmol/L), and a free T3 of 4.08 pmol/L (reference range: 3.10–6.80 pmol/L). The thyroid autoimmunity study was negative: antithyroperoxidase antibodies <14 IU/mL, antithyroglobulin antibodies 16 IU/mL (<115 IU/mL), and TRAb < 0.80 U/L. The patient had no previous history of thyroid dysfunction, thyroid nodules, or exposure to radiation. Family history of thyroid disease was unremarkable. On examination, there were no visible signs of cervical inflammation. The patient had mild tenderness on thyroid palpation and no fluctuation in the region. Thyroid ultrasound excluded the presence of thyroid nodules and showed a globally heterogeneous pattern with diffusely decreased vascularization of the gland parenchyma. A diagnosis of destructive thyrotoxicosis was assumed. The adrenergic symptoms were managed with a beta‐blocker.

**Figure 2:**
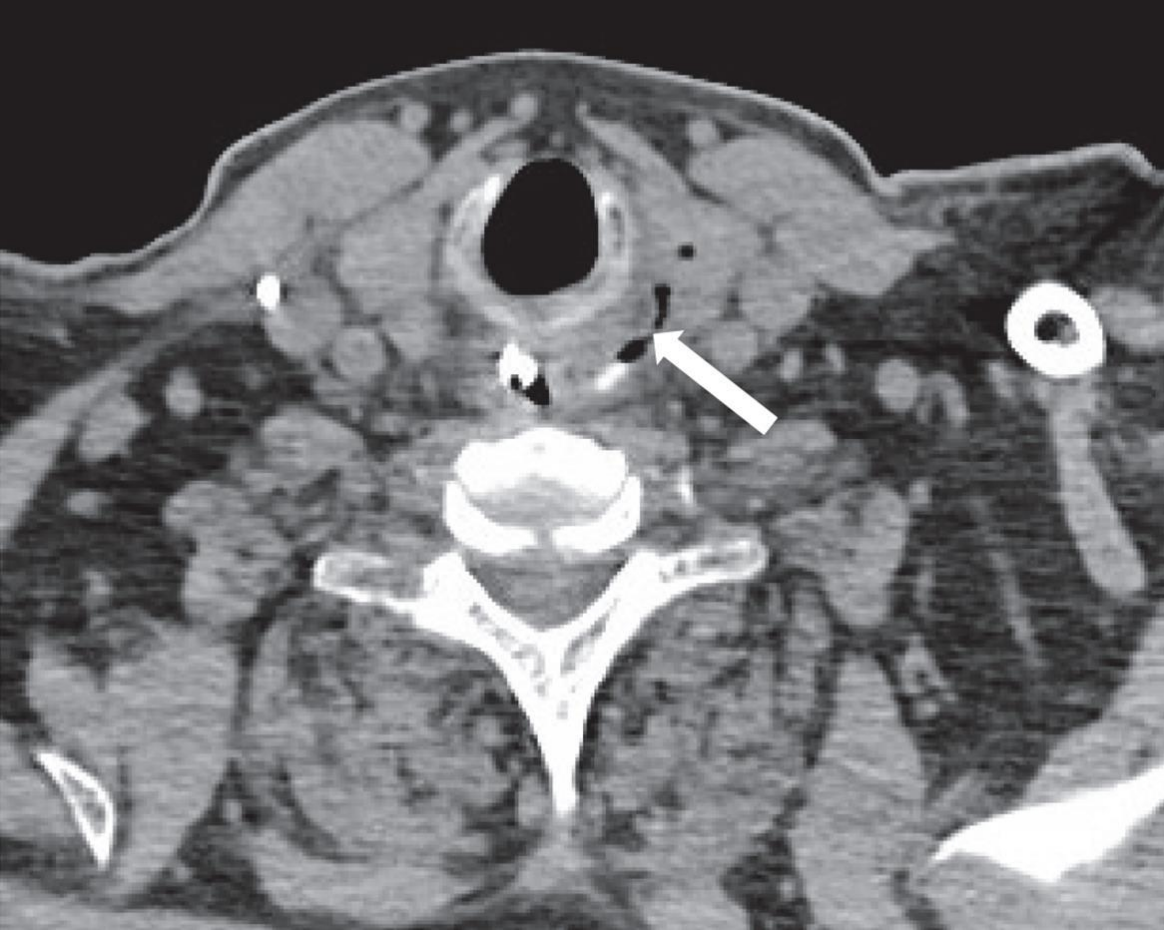
Axial image of neck CT with oral contrast (1 mm slice thickness), showing contrast extravasation at the cricopharyngeal plane within a filiform air tract extending to the posterior border of the left thyroid lobe (arrow).

A neck CT with oral contrast performed 5 days after the foreign body extraction (shown in Figure 2) revealed contrast extravasation at the cricopharyngeal plane, more specifically in the region where the foreign body was previously lodged. The oral contrast partially filled a filiform air tract extending to the posterior border of the left thyroid lobe, measuring 22 mm in length, which confirmed a small perforation at that level with low output.

**Figure 3:**
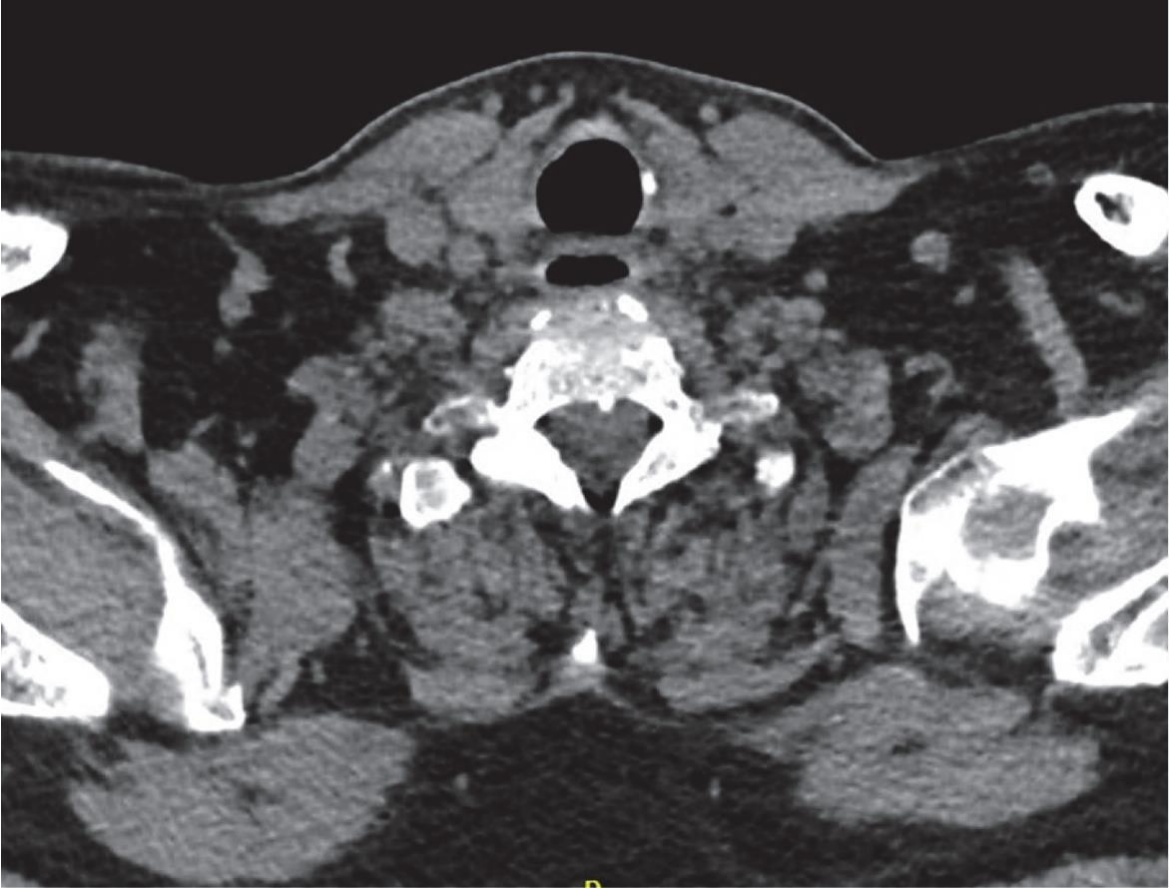
Axial image of neck CT with oral contrast (1 mm slice thickness), showing resolution of thyroid pneumatosis and no contrast extravasation.

The case was discussed with a multidisciplinary team, including Otolaryngology, General Surgery, and Endocrinology: a conservative approach was decided, and the patient was transferred to the Otolaryngology ward. He was maintained on an enteral diet via nasogastric tube until there was no evidence of contrast extravasation on follow‐up neck CT with oral contrast, 2 weeks postintervention. This exam revealed resolution of thyroid pneumatosis (Figure 3).

The patient progressed favorably. Oral intake was restored, cervical discomfort resolved, and inflammatory markers improved. He was discharged and subsequently followed up in both Otolaryngology and Endocrinology clinics. One month after the diagnosis of thyroid pneumatosis, laboratory results showed a TSH of 6.90 mIU/mL, free T4 of 12.9 pmol/L, and free T3 of 4.15 pmol/L. Two months later, thyroid function had fully normalized (TSH 2.44 mUI/mL, free T4 13.6 pmol/L, and free T3 4.58 pmol/L), without thyroid hormone supplementation.

## 3. Discussion/Conclusion

We describe a rare case of thyroid pneumatosis. To the best of our knowledge, this is the first case of thyroid pneumatosis described not related with thyroid FNA biopsy [1].

In our patient, thyroid pneumatosis likely resulted from superior esophagus perforation due to fishbone ingestion and subsequent surgical extraction, leading to a fistula between the left thyroid lobe and the superior esophagus at the cricopharyngeal plane.

Fishbones are the most common esophageal foreign bodies among adults, and their ingestion may be associated with a risk of esophageal perforation [2]. Some of the most commonly reported complications include retropharyngeal abscess, mediastinitis, migration of the foreign object into the neck’s fascial spaces, arterial‐esophageal fistula leading to hemorrhage, pneumomediastinum, sepsis, and pneumothorax [2, 3].

In the cervical region, air or gas outside the aerodigestive tract is pathological, and various mechanisms may be implicated: traumatic disruption of cutaneous or mucosal barriers (e.g., disruption of pharyngeal, tracheal, and esophageal integrity), infectious process with gas‐forming microorganisms, or increased pressure on air pathways [4, 5].

Our case suggests that foreign body traumatic perforation of the esophagus led to air dissemination from the esophageal lumen to the soft tissues in the cervical visceral space, a compartment bound by the visceral layer of the deep cervical fascia (pretracheal fascia), enclosing the thyroid and parathyroid glands, larynx, trachea, pharynx, and esophagus [4, 5].

Infection of the thyroid gland by gas‐producing microorganisms appears unlikely in this case. Acute suppurative thyroiditis is a rare condition, most commonly associated with immunocompromised individuals. The second most frequent predisposing factor is a pyriform sinus fistula, a congenital remnant of the branchial apparatus [6]. Neither of these risk factors was identified in our patient. Although abscess cultures could have yielded valuable information, in helping to exclude infection by gas‐producing microorganisms, they were not obtained at the time of surgical drainage. Other findings, however, argue against the likelihood of thyroid infection. Clinically, acute suppurative thyroiditis typically presents with prominent anterior neck pain [6], which was notably absent. There was no fever at the time of pneumatosis, and physical examination revealed no palpable masses, significant tenderness, or fluctuance to suggest a localized collection. Thyroid pneumatosis was observed only after the initiation of antibiotic therapy—at a time when the inflammatory markers were declining. Imaging studies showed no thyroid enlargement or abscess formation but did demonstrate a clear fistulous tract extending from the esophagus to the thyroid parenchyma, allowing escape of air from a gas‐containing viscus into the thyroid gland.

In our case, thyroid pneumatosis caused destructive thyroiditis, characterized by a triphasic course: thyrotoxicosis, hypothyroidism, and return to normal thyroid function [7–9]. Initially, thyroid cell damage leads to hormone leakage, causing thyrotoxicosis with elevated free T4 and suppressed TSH. Free T4 levels are increased preferentially compared to the total T3, in contrast to other forms of thyrotoxicosis [10]. As inflammatory process persists, the thyroid becomes depleted of hormones, leading to hypothyroidism [7–9]. Thyroid ultrasonography revealed diffuse heterogeneity and decreased vascularization, suggestive of destructive thyroiditis, rather than the increased flow characteristic of Graves’ disease [7, 11].

The case was reviewed by a multidisciplinary team who opted for a conservative approach. The patient had a favorable outcome, with resolution of thyroid pneumatosis within 2 weeks. Thyroid function returned to normal within 2 months after the diagnosis. The patient was initially treated with beta‐blockers to control adrenergic symptoms. In cases of destructive thyroiditis, antithyroid medications are not indicated since the thyroid hormone production is not increased [7, 8].

To the best of our knowledge, this is the first case that describes destructive thyroiditis related to thyroid pneumatosis.

The only previously documented case of thyroid pneumatosis was described by Li et al. [1]. It involved a 44‐year‐old woman with papillary thyroid carcinoma and occurred after an ultrasound‐guided FNA biopsy, where the needle punctured the cervical trachea, causing an air leak [1]. A CT performed 2 days later revealed thyroid pneumatosis. The patient had cervical discomfort, but no thyroid dysfunction was reported. A watchful waiting approach was taken, and a follow‐up CT 2 days later showed significant gas reabsorption [1].

Thyroid pneumatosis should be considered a potential rare complication following esophageal perforation and may present with destructive thyroiditis. Importantly, thyroiditis can arise from mechanical insults such as pneumatosis, even in the absence of overt infection. Given this potential risk, we propose that patients with esophageal perforations adjacent to the thyroid bed should undergo TSH and free T4 monitoring for a minimum of 4 weeks to facilitate detection and management of thyroid dysfunction. Our recommendation focuses on the surveillance of patients with radiologic signs of thyroid parenchymal involvement, as these individuals are at higher risk for developing thyroid dysfunction. Routine monitoring may also be considered in cases of perforation in close proximity to the thyroid without radiological evidence of thyroid involvement, depending on clinical judgment.

## Author Contributions

Every person listed as an author made a significant contribution to the writing and revision of this case.

## Funding

This work did not receive any specific grant from any funding agency in the public, commercial, or not‐for‐profit sector.

## Disclosure

Every person listed as an author approved the final version for submission for publication.

## Ethics Statement

Written informed consent has been obtained from the patient for publication of the case report and accompanying images.

## Conflicts of Interest

The authors declare no conflicts of interest.

## Data Availability

The data that support the findings of this study are available upon request from the corresponding author. The data are not publicly available due to privacy or ethical restrictions.

## References

[1] Li H. , Chen W. , and Xu H. , et al.A First Report of Thyroid Pneumatosis as a Complication of Ultrasound-Guided Thyroid Biopsy, Current Medical Imaging. (2023) 20, 10.2174/1573405620666230405095428. 37038670

[2] Loh K. S. , Tan L. K. , Smith J. D. , Yeoh K. H. , and Dong F. , Complications of Foreign Bodies in the Esophagus, Otolaryngology–Head and Neck Surgery. (2000) 123, no. 5, 613–616, 10.1067/mhn.2000.110616. 11077351

[3] Aronberg R. M. , Punekar S. R. , Adam S. I. , Judson B. L. , Mehra S. , and Yarbrough W. G. , Esophageal Perforation Caused by Edible Foreign Bodies: A Systematic Review of the Literature, The Laryngoscope. (2015) 125, no. 2, 371–378, 10.1002/lary.24899, 2-s2.0-84921651519. 25155167

[4] Frias Vilaça A. , Reis A. M. , and Vidal I. M. , The Anatomical Compartments and Their Connections as Demonstrated by Ectopic Air, Insights into Imaging. (2013) 4, no. 6, 759–772, 10.1007/s13244-013-0278-0, 2-s2.0-84889560618. 24065628 PMC3846937

[5] Maunder R. J. , Pierson D. J. , and Hudson L. D. , Subcutaneous and Mediastinal Emphysema. Pathophysiology, Diagnosis, and Management, Archives of Internal Medicine. (1984) 144, no. 7, 1447–1453, 10.1001/archinte.1984.00350190143024, 2-s2.0-0021281465. 6375617

[6] Lafontaine N. , Learoyd D. , Farrel S. , and Wong R. , Suppurative Thyroiditis: Systematic Review and Clinical Guidance, Clinical Endocrinology. (2021) 95, no. 2, 253–264, 10.1111/cen.14440. 33559162

[7] Ross D. S. , Burch H. B. , and Cooper D. S. , et al.American Thyroid Association Guidelines for Diagnosis and Management of Hyperthyroidism and Other Causes of Thyrotoxicosis, Thyroid. (2016) 26, no. 10, 1343–1421, 10.1089/thy.2016.0229, 2-s2.0-84991406672. 27521067

[8] Wiersinga W. M. , Poppe K. G. , and Effraimidis G. , Hyperthyroidism: Aetiology, Pathogenesis, Diagnosis, Management, Complications, and Prognosis, The Lancet Diabetes & Endocrinology. (2023) 11, no. 4, 282–298, 10.1016/S2213-8587(23)00005-0. 36848916

[9] Samuels M. H. , Subacute, Silent, and Postpartum Thyroiditis, Medical Clinics of North America. (2012) 96, no. 2, 223–233, 10.1016/j.mcna.2012.01.003, 2-s2.0-84858768971. 22443972

[10] Carlé A. , Knudsen N. , and Pedersen I. B. , et al.Determinants of Serum T4 and T3 at the Time of Diagnosis in Nosological Types of Thyrotoxicosis: A Population-Based Study, European Journal of Endocrinology. (2013) 169, no. 5, 537–545, 10.1530/EJE-13-0533, 2-s2.0-84885159943. 23935127

[11] Kahaly G. J. , Bartalena L. , Hegedüs L. , Leenhardt L. , Poppe K. , and Pearce S. H. , 2018 European Thyroid Association Guideline for the Management of Graves’ Hyperthyroidism, European Thyroid Journal. (2018) 7, no. 4, 167–186, 10.1159/000490384, 2-s2.0-85051952417. 30283735 PMC6140607

